# Long-Term Analysis of Elite Basketball Players’ Game-Related Statistics Throughout Their Careers

**DOI:** 10.3389/fpsyg.2019.00421

**Published:** 2019-02-27

**Authors:** Jorge Lorenzo, Alberto Lorenzo, Daniele Conte, Mario Giménez

**Affiliations:** ^1^Sport Science Department, Universidad Politécnica de Madrid, Madrid, Spain; ^2^Institute of Sport Science and Innovations, Lithuanian Sports University, Kaunas, Lithuania

**Keywords:** evolution, statistics, tendencies, career, team sports, professional

## Abstract

The aim of the present study was to analyze the changes of game-related statistics in expert players across their whole sports careers. From an initial sample including 252 professional basketball players competing in Spanish first division basketball league (ACB) in the 2017–2018 season, 22 met the inclusion criteria. The following game-related statistics were studied: average points, assist, rebounds (all normalized by minute played), 3-point field goals percentage, 2-point field goals percentage, and free throws percentage per season. Each variable was individually investigated with a customized excel spreadsheet assessing individual variations and career trends were calculated. The results showed a positive trend in most of the investigated players in assists (91% of cases) and free throw percentages (73% of cases). Similar percentages of positive and negative trends were observed for all the other game-related statistics (range: 41–59% for negative and positive, respectively). In conclusion, an increase in assist and free throw performance was shown in the investigated players across their playing career. This information is essential for basketball coaches suggesting the use of most experienced players in the final moments of the game.

## Introduction

Basketball is a team sport characterized by the execution of series of skills in multiple situations occurring across the game. In particular, game-related statistics are fundamental and their level might depend on the players’ characteristics and training experience. Most of the game related statistics depends on multifactorial variables (i.e., offensive and defensive tactics) determining a complex dynamic system during games, which is difficult to control in its totality. The use of performance analysis in sport with the determination of the most important game related statistics during the game aims to improve the team performance, increasing the knowledge of the performance of each player. Specifically, game-related statistics are key tools for basketball coaches providing reliable information about teams’ performance such as those distinguishing between successful and unsuccessful teams. Previous investigations widely studied the game-related statistics mostly assessing team performance in order to determine the most valuable players and the importance of certain positions such as guards, forward and centers (e.g., Sampaio et al., 2006a), to evaluate the impact of rule changes (e.g.; Gómez et al., 2006a; Ibáñez et al., 2018), the effect of home advantage (e.g.; Carron et al., 2005; Pollard, 2008; Watkins, 2013), the importance of starters and bench players regarding their contribution to the game (e.g.; Sampaio et al., 2006b), the scoring strategies differentiating between winning and losing teams in women’s basketball FIBA Eurobasket (e.g.; Conte and Lukonaitiene, 2018). It is important to note that in basketball several game related statistics have been used, while only some of them were deemed fundamental. Previous discriminant analyses quantitatively determined the team performance indicators (TPI), identified as a variable able to define the most important aspect of performance (Hughes and Bartlett, 2002) and compare different leagues (Sampaio and Leite, 2013), which most affect the game outcome (Gomez et al., 2008; Ibánez et al., 2008). In particular, Yu et al. (2008), established a list of the most influential TPI’s (Technical Performance Indices) such as points per game (PPG), field goals made (FGM), rebounds, assists, turnovers, blocks, fouls, and steals. Sampaio et al. (2013) included also free throws as an important technical performance indicator. The TPIs with the most impact on the outcome of a season in Spanish first division (ACB) teams were shooting percentage (both 2-point and 3-point percentage), assists and rebounds (García et al., 2013; Gómez et al., 2008). However, to the best of our knowledge, no previous investigations assessed players’ individual game related statistics across a long period of time. Indeed, players’ experience might play a fundamental role in improving players’ game related statistics effectiveness. Therefore, studies addressing this topic are warranted.

The performance of a player across his career might play a fundamental role in distinguishing between elite and non-elite players. Indeed, acquiring playing experience, players could have a better performance due to the demand of basketball game to perform complex actions that require high anticipatory skills in difficult situations. Indeed, these high anticipatory skills can be translated into scoring and passing related variables concerning about game-related statistics (Sampaio et al., 2015), and therefore they become an important variable deeming further analysis in basketball. In fact, elite players perceive better their environmental information and are capable of adapting their behavior accordingly and consequently perform better compared to other non-elite players (Aglioti et al., 2008). Therefore, playing experience might be essential in increasing players’ anticipatory skills and consequently their game performance.

It has been previously showed that performance slowly decrease after reaching the peak period of the player career (Baker et al., 2013). In basketball, Baker et al. (2013), found that the typical basketball career lasts about 11 years, with the longest career studied being 23 years of playing at an elite level. However, it is not clear the performance changes across players career, and their trend (i.e., positive or negative) calling for further studies in this area. Therefore, the aim of this study was to descriptively analyze TPI changes throughout the career of expert basketball players, assessing the possible performance trend.

## Materials and Methods

### Participants

From an initial sample of 252 professional basketball players competing in ACB, 22 players (9 backcourt and 13 frontcourt) were selected for this study based on the following inclusion criteria determined by a group of experts, who were identified according to Swann et al. (2015) guidelines: (a) male players, currently playing in the ACB league in the season 2017–2018; (b) to have a minimum playing experience of 10 years (including only season in which they effectively played) in the first division of any country with at least an average of 25% of number of games and minutes played per season; (c) to possess a minimum of 5 years playing experience in first division of any league amongst the top 30 countries in the FIBA Ranking (at February 28, 2018); (d) to have played at least 75% of their professional careers in any country’s first division league, consequently no years played in lower division leagues were analyzed. The aim of these criteria was to ensure the highest quality of the sample for expert players with a solid number of games and minutes played each season (Swann et al., 2015).

### Procedure

The databases used to obtain the game related statistics of each season for the studied players were the ACB official web page^1^ for any season played in the ACB league, and the RealGM website^2^, or the official ACB guide released by the Spanish Basketball Association for any season played outside Spain. These databases are normally used in studies related with basketball, and basketball statistics and are considered valid and reliable (Gómez et al., 2018).

The following game-related statistics for each season were recorded and analyzed: average points, assist, rebounds, 3-point field goals percentage, 2-point field goals percentage and free throws percentage per season. The variable point, assists and rebounds were normalized by minute played with the following formula (example for points scored: mean seasonal points scored/mean seasonal minute played ^∗^40 min). All the data for these game-related statistics, for every season and every player included in this study were storage in a database and once they were used for the statistical analysis.

### Statistical Analysis

**FIGURE 1:**
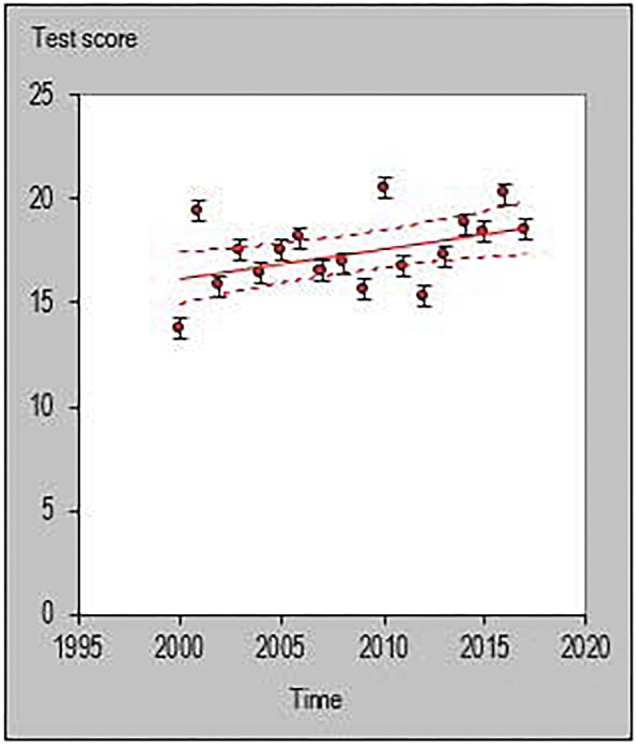
Individual trend of one participant for average points per season normalized by minute.

**FIGURE 2:**
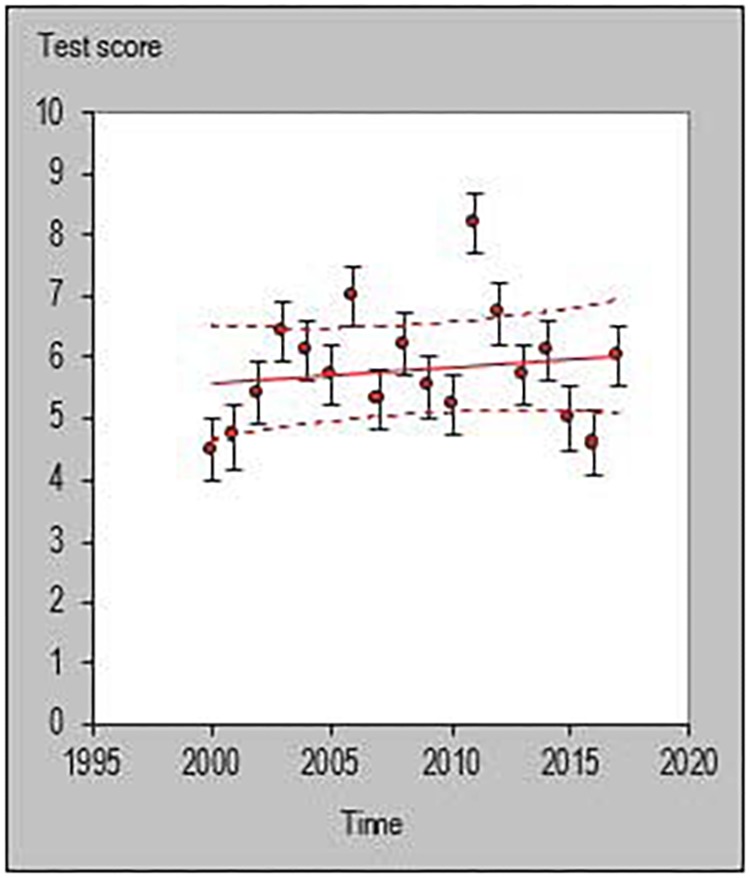
Individual trend of one participant for average rebounds per season normalized by minute.

**FIGURE 3:**
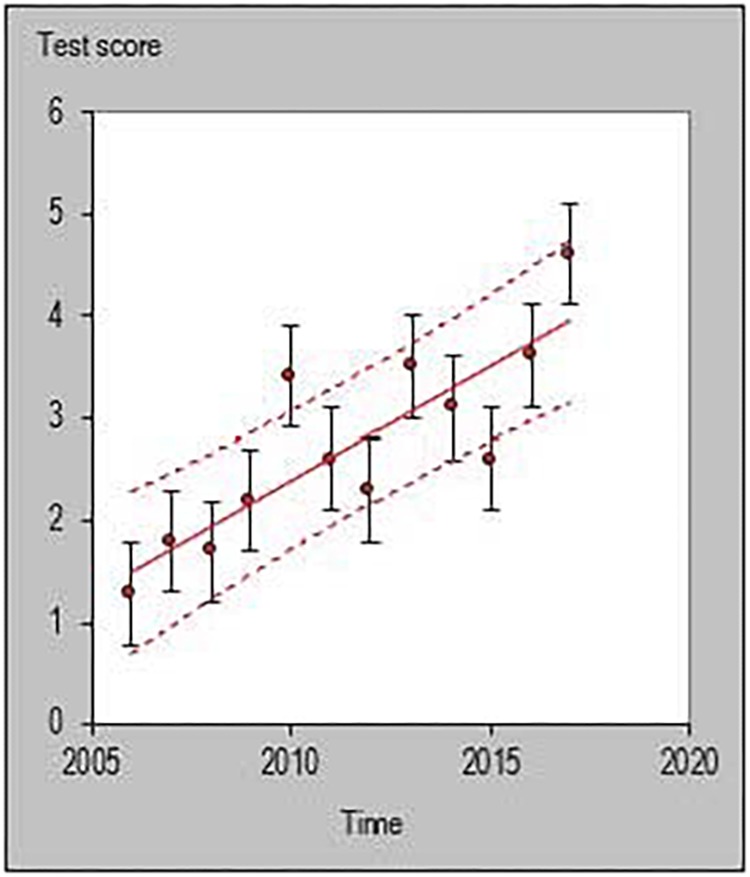
Individual trend of one participant for average assists per season normalized by minute.

**FIGURE 4:**
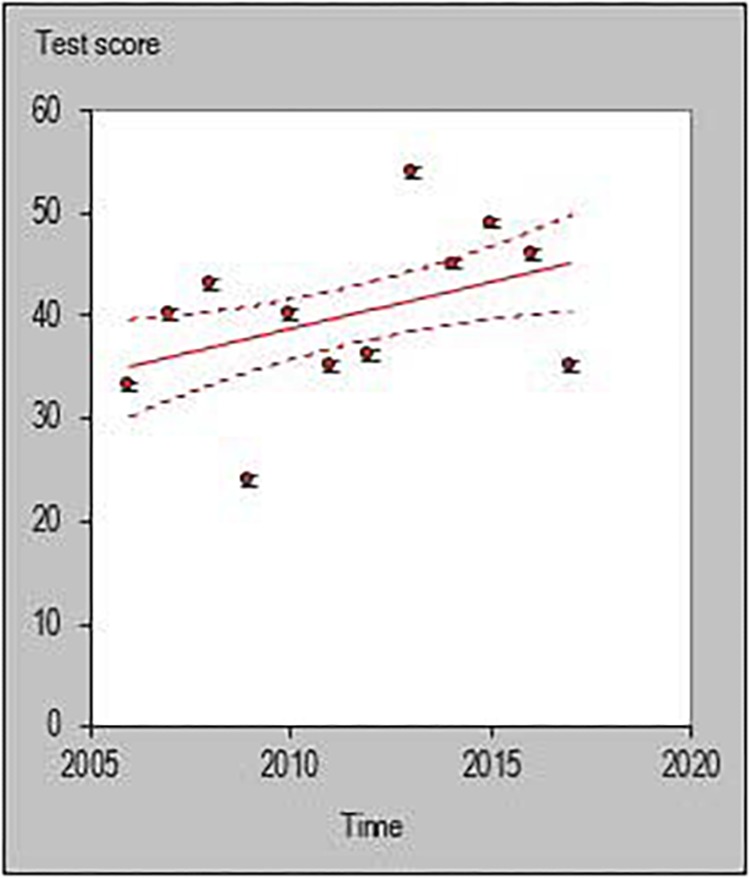
Individual trend of one participant for 3-point percentage per season.

**FIGURE 5:**
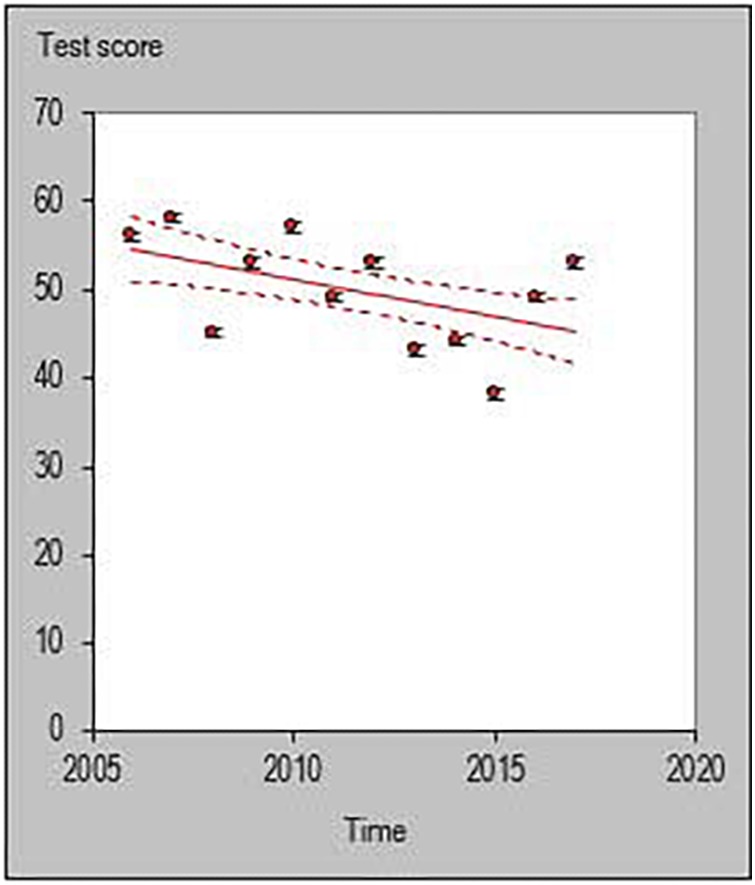
Individual trend of one participant for 2-point percentage per season.

**FIGURE 6:**
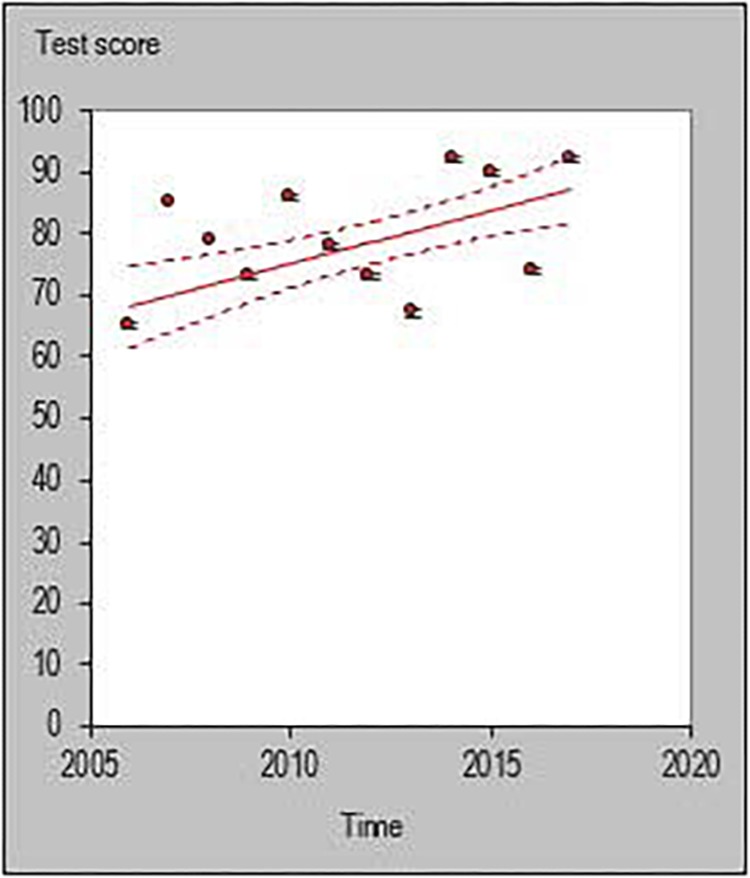
Individual trend of one participant for free throw percentage per season.

All statistical analyses were performed with a customized excel spreadsheet specifically developed to monitor individual changes and trends in a rigorous quantitative way (Hopkins, 2017). Recently, this excel spreadsheet has been adopted to assess individual changes in team sports (Siahkouhian and Khodadadi, 2013; Loturco et al., 2017; Colyer et al., 2018; Hurst et al., 2018) and specifically in basketball (Pliauga et al., 2018). This statistics approach could be used as a possible alternative to previously used methodologies such as the ANOVA factor (Yu et al., 2008) or the Jonckheere–Terpstra test (Leite and Sampaio, 2012). The individual trends across playing career for each investigated player were then quantified and the percentage of players documenting a positive, negative or steady (when the result is zero) slope were calculated using the following formula y = m⋅x+n. Figure 1–6 are an example of the individual points and trendlines obtained via the Hopkins spreadsheet and that were later analyzed.

## Results

The mean slope for each performance indicator and the number of cases in which the slope was positive, steady, or negative are shown in Table 1. Results revealed that most of the players have a positive trend in assists (91% of the cases) free throws (73% of the cases), and 3-point percentage although with a lower value (59%). Conversely, there were no differences of positive and negative trends reported for the other investigated parameters (Table 1).

**Table 1:** Mean slope and number of cases of each variable.

	Expert players (*N* = 22)				
	Positive	Mean slope	Steady	Negative	Mean slope
Points	10	0,24	0	12	-0,37
Assists	20	0,15	1	1	-0,1
Rebounds	10	0,12	0	12	-0,07
3-point %	13	0,86	1	8	-0,53
2-point %	10	0,37	0	12	-0,49
Free throw %	16	0,95	0	6	-0,35


## Discussion

The aim of this study was to analyze the trends TPIs throughout the career of expert basketball players. The results revealed that assists and free throws were the two TPIs mostly showing a positive trend during players’ careers. Specifically, the 91% of the studied players have a positive tendency in assists, with a mean slope of 0.15, and 73% of them have a positive tendency in free throws, with a mean slope of 0.95. Also, 59% of the players increase their 3-point percentage, but this result might have been influenced by the fact that more frontcourt than backcourt players met our inclusion criteria.

Basketball is a sport where situations change quickly and continuously as a result of the combination of factors such as the position of opponents in the field and their tactical behavior, the position of the ball and the timing of the offensive movements (Altavilla and Raiola, 2014). Therefore, players are required to decide an appropriate response with a proper timing and executing it in a correct spacing. Often, players are subject to defensive pressure and the more skilled and experienced players might be able to anticipate events and perform unhurried actions as a result of their improved ability to “read the game” (Sampaio et al., 2004). In this context, executing a successful pass (i.e., assist) assume a fundamental importance in basketball. Indeed, when analyzing the mechanism of this technical action, the assist requires a combination of good decision making in court, coordination, anticipation, timing, and a good execution (Melnick, 2001; Gómez et al., 2006b). Previous research demonstrated that assists and free throw percentage are two of the most factors to win a game (Dias, 2007; Gómez and Lorenzo, 2007; Sampaio et al., 2015). Moreover, Sampaio et al. (2004), suggested that assists are indicators of players’ maturity and experience, increasing in number as the player gets a better ability to read the game due to the years of playing experience. The results of our investigation highlighted supporting results, since most of the investigated players increased their assist performance across their playing career. This information seems essential for basketball coaches, who can rely on the performance of more experienced basketball players characterized by a better tactical awareness in order to execute successful passes and increase the scoring possibilities during the game. Indeed, Melnick (2001) showed a positive correlation between number of assists of a team and a better win-loss record through a season.

Free throws have also been demonstrated to be performance indicators differentiating between winning and losing teams in particular in close games (Ibánez et al., 2008; Conte et al., 2018; Gómez et al., 2018). Therefore, it was expected that players increasing their experience and possibly assuming a leadership and fundamental role in their team would increase their free throw performance during their career. Accordingly, our results demonstrated an increased trend across players’ career for free throws and therefore possibly increasing their teams’ possibility to be successful. In this sense, experience accumulated in games and practices is the most crucial factor for developing expertise in one aspect (Gómez et al., 2018). An increase in the percentages of free throws can be associated with the fact that players have already mastered the shooting during their years of training. Interestingly, a previous investigation showed that free throws shooting trajectories are more efficient and possess a lower variability in more experienced players compared to less experienced players (Button et al., 2003). The practical application of our result is that coaches should favor the participation of most experienced players in last minutes of close games, when usually there are higher number of fouls generating free throws opportunities.

Other variables such as points, rebounds, and 2-point percentage did not show any trend increase across the players’ career. A possible reason for this finding is that these variables might be more influenced by physical factors (i.e., strength, power, and fitness), which showed a decrease during the lifespan (Horton et al., 2008). Even though experienced players compensate this decrease in their physical abilities with a better understanding of the games’ tactical aspects, better timing and spacing and better decision-making abilities, it seems not enough to show a positive trend according to the results of our investigation.

Although this investigation provides basketball coaches with useful information, some limitations should be mentioned. Firstly, the results might have been influenced by some confounding factors such as injuries across the season, the playing status (i.e., starting vs. bench players), economical aspects such as players’ contracts and players and/or coaching staffs changing teams during investigated period. Therefore, future studies are warranted in order to overcome these limitations possibly controlling these factors. However, to the best of our knowledge, this investigation provides the first evidence about the individual trend in players’ performance across their playing career and notably increase the knowledge in this field. Moreover, further studies should be designed in order to assess players’ individual season-by-season changes across their playing career.

## Conclusion

The results of this investigation suggest that as the players acquire years of experience in first division elite teams, their assists per game and free throw percentage increase. Conversely, other game-related statistics such as points, rebounds, 3-point percentage, and 2-point percentage showed both positive and negative trends in the investigated players resulting in a high between players variability. Finally, further research is required in this field using an individualized approach to increase the knowledge about players’ performance across their playing career.

## Author Contributions

All authors listed have made a substantial, direct and intellectual contribution to the work, and approved it for publication.

## Conflict of Interest Statement

The authors declare that the research was conducted in the absence of any commercial or financial relationships that could be construed as a potential conflict of interest.

## Footnotes

1 http://www.acb.com

2 https://basketball.realgm.com
